# Effects of Non-thermal Ultrasound on a Fibroblast Monolayer Culture: Influence of Pulse Number and Pulse Repetition Frequency

**DOI:** 10.3390/s21155040

**Published:** 2021-07-25

**Authors:** Silvia Ronda Peñacoba, Mar Fernández Gutiérrez, Julio San Román del Barrio, Francisco Montero de Espinosa

**Affiliations:** 1 ITEFI-CSIC, Institute of Physics and Communication Technologies, 28006 Madrid, Spain; francisco.montero@csic.es; 2 ICTP-CSIC, Institute of Polymer Science and Technology, 28006 Madrid, Spain; ictf339@ictp.csic.es (M.F.G.); jsroman@ictp.csic.es (J.S.R.d.B.); 3 CIBER, Carlos III Health Institute, 28029 Madrid, Spain

**Keywords:** ultrasound, cell mechanotransduction, fibroblasts culture

## Abstract

Despite the use of therapeutic ultrasound in the treatment of soft tissue pathologies, there remains some controversy regarding its efficacy. In order to develop new treatment protocols, it is a common practice to carry out in vitro studies in cell cultures before conducting animal tests. The lack of reproducibility of the experimental results observed in the literature concerning in vitro experiments motivated us to establish a methodology for characterizing the acoustic field in culture plate wells. In this work, such acoustic fields are fully characterized in a real experimental configuration, with the transducer being placed in contact with the surface of a standard 12-well culture plate. To study the non-thermal effects of ultrasound on fibroblasts, two different treatment protocols are proposed: long pulse (200 cycles) signals, which give rise to a standing wave in the well with the presence of cavitation (I_SPTP_ _max_ = 19.25 W/cm^2^), and a short pulse (five cycles) of high acoustic pressure, which produces a number of echoes in the cavity (I_SPTP_ = 33.1 W/cm^2^, with P_max_ = 1.01 MPa). The influence of the acoustic intensity, the number of pulses, and the pulse repetition frequency was studied. We further analyzed the correlation of these acoustic parameters with cell viability, population, occupied surface, and cell morphology. Lytic effects when cavitation was present, as well as mechanotransduction reactions, were observed.

## 1. Introduction

Ultrasound (US) has been used in rehabilitation and physiotherapy since the 1930s [1,2], as well as in surgery and oncology, to treat injured tissues [3,4], kidney stones [5], and tumors [6,7], or to promote healing by increasing the therapeutic rate of other treatments [8,9]. Exposure to US is known to produce a variety of biological effects in tissues, which can be grouped into thermal and non-thermal effects. The heating of tissues that occurs is due to the absorption of the sound wave [10], but there is also a mechanical interaction between the pressure wave and the medium, which gives rise to mechanisms such as radiation force, streaming, and cavitation. Despite evidence that it can produce physical changes in tissues, there is still controversy in the literature about the use of US in clinical practice, particularly with regards to its efficacy [11,12]. Therefore, in vitro studies are essential in order to understand how this type of radiation can be optimized for better results.

A limitation in the review of the available literature regarding the application of US in in vitro cultures is that no standard sonication dosimetry parameters have been used in these studies. Contradictory results have been found, where the reason could be that the characterization of the acoustic field in the experiment has been incomplete or, in some cases, ignored (e.g., relating to stationary wave formation or the existence of hydrostatic pressure [13]). For this reason, it is necessary to analyze and quantify the acoustic parameters in the experimental conditions used in each case.

One of the most widely used in vitro experiments with US consists of the study of the evolution of a cell monolayer on a liquid culture, where the ultrasonic transducer is in contact with the culture plate. Acoustic adaptation is achieved by means of a hydrogel coupling layer. This configuration features an almost perfect reflective air–liquid interface (Г ≈ 0.9994) and, therefore, stationary waves are created in the liquid column if the acoustic wave pulse is longer than twice the liquid’s height. In such a configuration, it is difficult to obtain reproducible results, as small variations in the volume of the liquid can cause strong changes in the amplitude of the acoustic pressure at the cell culture level, as has been shown by Hensel K et al., who assumed that the transducer had an acoustic aperture similar to that of an ideal piston [13].

In this work, two US protocols were used to study their non-thermal effects in a single-layer fibroblast culture. Fibroblasts were chosen as the biological target of choice because they are the main cells in connective tissue and play a fundamental role in the synthesis of the extracellular matrix (ECM), which supports the musculature. The purpose of this study is to contribute to designing a new treatment protocol for chronic diseases of soft tissues (i.e., muscles and tendons). For this reason, fibroblast cells appear to be the most suitable cells to test the intensity threshold intervals between harmless (and, therefore, useful for rehabilitation) and ablative (useful in surgery and oncology) US exposure.

We are interested in studying the non-thermal effects of US. Therefore, sonication protocols with a maximum duty cycle (DC) of 10% were used in the tests in order to avoid heating of the medium due to absorption [10]. The non-thermal effects of US are associated with pressure wave interactions with the cells. Three mechanical actions can be distinguished: (i) Radiation force action; (ii) pressure interaction with the cell membrane mechanosensors; and (iii) cavitation, which is stable if the bubble just oscillates and inertial if it collapses. Cavitation effects in in vitro cultures have been reported previously [14,15,16,17,18,19,20,21,22]. Changes produced in the cells include membrane disruption, opening of reversible or irreversible pores, ion channels, and toxicity. Effects in the cells below the cavitation threshold, including proliferation, substance uptake, cell signaling, or motility increase, have also been reported [23,24,25,26,27,28,29]. 

This paper is the first to present an in vitro experiment that studies the effect of the sonication parameters of short pulses with pressure levels up to 1 MPa, in order to apply the results in focused non-thermal medium intensity ultrasonic physiotherapy treatments for tendinopathies and trigger point diseases. For this purpose, two acoustic field configurations were studied: stationary wave and multiple reflections of a pulse. Previous works have shown that a lower acoustic intensity is needed to damage the cells when a stationary field is created in a liquid, owing to the presence of cavitation [30]. In our work, sono-spectroscopy is used to reveal the presence of stable cavitation.

## 2. Materials and Methods

### 2.1. Ultrasound Treatment Set-Up

**Figure 1:**
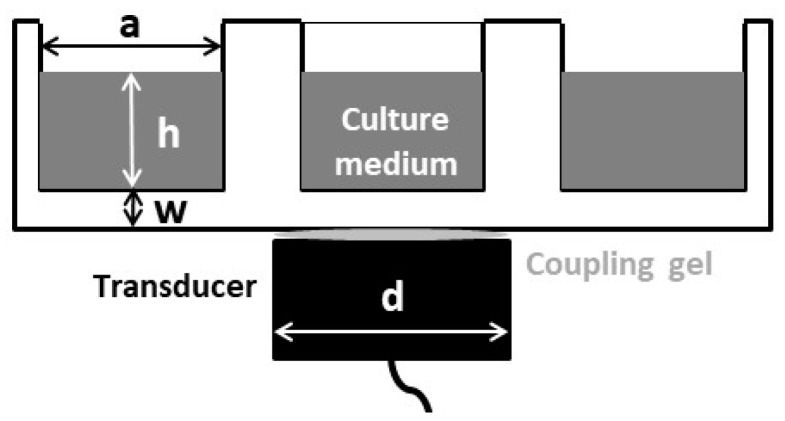
Schematic diagram of the ultrasound (US) exposure set-up. Fibroblasts are attached on the well, at the bottom of the liquid column. a = 22.2 mm, h ≈ 10 λ (7.7 mm), w = 1.22 mm, d = 25 mm.

A schematic of the US exposure conditions for the in vitro experiments is shown in Figure 1. The transducer was a single 25 mm diameter PZT-4 air-backed piezoceramic disc without a matching layer. 

The nominal mechanical resonance frequency was 1.9 MHz. This frequency was selected as it is intermediate between the two most commonly frequencies employed in physiotherapy US studies (TUS): 1 MHz and 3 MHz [3]. The electric excitation was delivered by a function generator (HP 8116 A), which was connected to a power amplifier (AR 100A250A). A thin hydrogel coupling layer (Aquasonic 100, Parker Lab. Inc., Fairfield NJ, USA) was used to couple the transducer to the culture well bottom. In all the experiments, the culture well was filled with 3 mL of culture medium, ensuring that the height of the liquid path was always the same. A positioning system was used to ensure reproducibility and parallelism in all the experiments.

### 2.2. Acoustic Parameters

The vibration amplitude distribution of the transducer at the working frequency was measured by laser vibrometry (Polytec OFV 5000). The acoustic pressure amplitude in the experimental conditions (with the well filled with liquid) was measured using a calibrated needle hydrophone (DAPCO 54389) with 0.6 mm of active diameter connected to an oscilloscope (Tektronix TDS210). The 2D acoustic field distribution at 0.5 mm to the well plate’s upper surface—where the cells grow—and the complete acoustic field distribution in the liquid volume were captured with a 3D mechanical displacement system controlled by a computer. The quantification of the maximum acoustic pressure value (P_max_), peak intensity (I_SPTP_), and energy density of the acoustic pulse ($\rho_{E}$) was carried out. The acoustic power emitted in free-field conditions was measured with a calibrated radiation force balance system implemented in our laboratory (I_SATP_) [31].

The same hydrophone and oscilloscope were also used to perform sono-spectroscopy measurements, in the same conditions as the sonication, in order to confirm/reject the presence of cavitation presence. For this purpose, signals were processed and the FFT absolute value was calculated (MATLAB^®^, MathWorks, Inc., Portola Valley, CA, USA), in order to analyze the harmonics present. The frequency response sensitivity of the hydrophone was considered.

Two different sonication protocols were used. The standing wave protocol used 200 cycles with a pulse repetition frequency (PRF) at 1 kHz and different pressure amplitudes (long pulse, LP). The short pulse treatment used five cycles of 1.01 MPa with PRF from 10 Hz up to 10 kHz (short pulse, SP).

### 2.3. Fibroblast Culture

Human dermal fibroblasts (HDFs) were used in the experiments (Innoprot^®^, Bizkaia, Spain). The cells were grown in an incubator at 37 °C under 5% ${\mathrm{CO}}_{2}$ in 75 cm^2^ polystyrene flasks filled with culture medium, as follows: Dulbecco’s modified Eagle’s medium enriched with 4500 mg/mL glucose (DMEM; Sigma, Madrid, Spain) supplemented with 10% fetal bovine serum (FBS), 200 mM L glutamine, 100 units/mL penicillin, and 100 µg/mL streptomycin and modified with HEPES (complete medium). When total confluence was reached, the cells were counted with a Countess Automated Cell Counter (Invitrogen^®^), to obtain 90,000 cells/mL. Then, the cells were attached in 12-well commercial polystyrene plates and grown as monolayers for 24 h. The plates were kept at 37 °C under 5% ${\mathrm{CO}}_{2}$, both prior to and after treatments.

### 2.4. Cell Viability Assay

Fibroblast viability was measured using an AlamarBlue^®^ assay, a redox indicator used to evaluate metabolic function, detected by fluorescence (460/630 em/ex) with a well plate reader (Biotek Synergy HT) [32]. Measurements at 24 h, 48 h, and 7 days after treatment were made. To compare the results, the treated fibroblasts and control groups were measured simultaneously and were subjected to the same method. The fluorescence value of each well plate was measured three times and the experiments were repeated at least twice.

### 2.5. Optic Microscopy Image Analysis 

After the 24 h post-treatment viability measurement, the culture well was tinted with crystal violet (CV, Sigma-Aldrich, St. Louis, MI, USA). Cells were fixed with glutaraldehyde at 2.5% for 10 min and washed with PBS (pH 7.4). After 15 min in darkness, excess CV was removed and the well plates were dried at an ambient temperature. This process allowed for visualization of the cells with an optical microscope (Nikon Eclipse 50i). Five images of the monolayer were taken, along the diameter of the well. This gave a set of a minimum of ten images of the state of each culture well after sonication for each experiment performed, considering that we repeated it at least twice for statistical reasons. The images were processed using the ImageJ^®^ software (NIH Image Inc.), in order to estimate the number of cells and the percentage of the surface occupied by fibroblasts. Cell morphology was also observed. Optical analysis of the images also revealed the presence of binucleated fibroblasts, which we were able to count. After 24 h, owing to the exponential growth of the culture, several overlapping cell sub-layers had formed, thus the binucleated cell count was no longer reliable.

## 3. Results

### 3.1. Ultrasound Field Characterization of the Culture Wells

**Figure 2:**
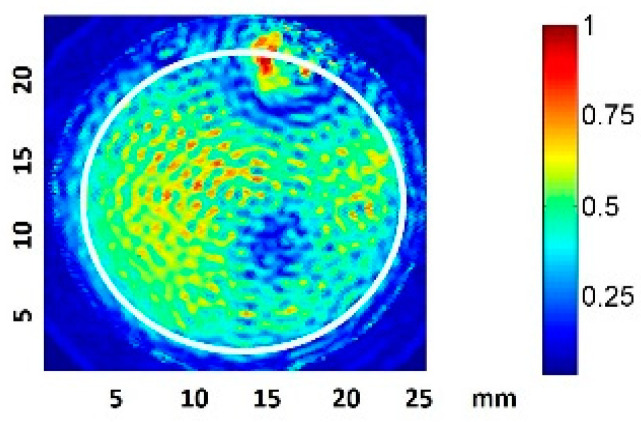
Normalized amplitude vibration of the transducer in the transversal direction, measured with laser vibrometry in air. The well plate surface contour is indicated in white.

The normalized transducer mechanical vibration perpendicular to the surface is shown in Figure 2. The contour corresponding to the well inner plate surface is represented by a white circumference. Some discontinuities, owing to how the wire was soldered, are evident. Throughout the surface, the amplitude lay in a range of 6 dB and the phase was constant, such that an ideal piston-like behavior can be expected. The effective radiating area of the transducer (ERA) was 3.5 cm^2^.

**Figure 3:**
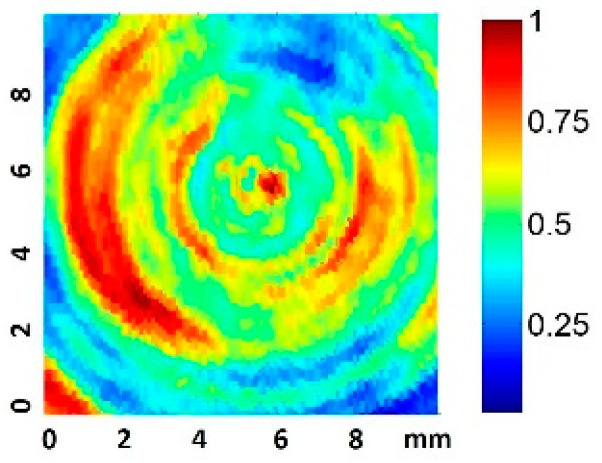
Normalized 2D Prms amplitude scan 0.5 mm from the well plate surface for a 200-cycle pulse.

We considered the root-mean-square pressure of the pressure, P_rms_, in order to take into account the acoustic pressure amplitude variations during the pulse duration. Some authors have suggested using this parameter when quantifying dosage, as P_rms_ is independent of the measurement set-up geometry, and hence could be used to predict the harmful threshold for cells under any in vitro experimental conditions [30]. A 2D scan of the normalized P_rms_ amplitude at 0.5 mm from the well bottom filled with culture medium was performed, as represented in Figure 3. The excitation was a 200-cycle pulse at 1.9 MHz.

The scan shows the predicted appearance of relative circular nodes and anti-nodes in the near zone [13]. The scan also presents pressure inhomogeneities (up to a factor of four) in zones correlated with the vibration inhomogeneity shown in the transducer vibration surface. This fact is considered in the analysis presented in the results. The acquisition of nine equidistant quantitative values at 0.5 mm along a complete diameter was also made, using a 10 V_pp_ driving voltage.

**Figure 4:**
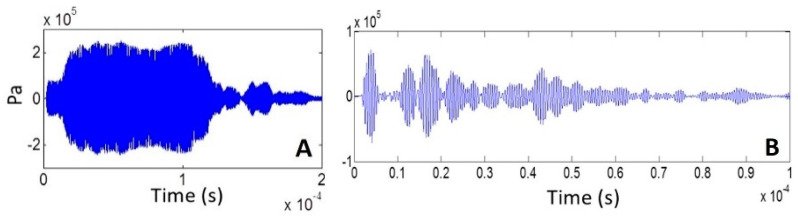
Acoustic signal in the center at 0.5 mm from the well bottom for (**A**) 200-cycle pulse and (**B**) 5-cycle pulse (10 Vpp transducer excitation).

The acoustic pressure signals at the center of the well bottom for the long and short pulses are shown in Figure 4A,B, respectively, from which standing constructive interference in the first case and multiple pulses in the second can be observed.

**Figure 5:**
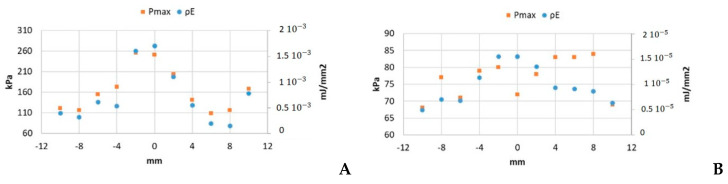
P_max_ and $\rho_{E}$ under 10 Vpp excitation for (**A**) 200-cycle pulse and (**B**) 5-cycle pulse.

Data obtained for the maximum acoustic pressure value (P_max_) and energy density ($\rho_{E}$) for the LP and SP excitation are shown in Figure 5A,B, respectively.

The ratio between the highest and the lowest values of P_max_ was 1.2 in the SP case (Figure 5B) and 2.3 for LP (Figure 5A). In the case of the energy density, the ratios were 2.3 for SP and 11.8 in the LP case.

Regarding to the literature devoted to US effects in cell liquid cultures, most of the authors have quantified the US dosage used in the experiments as indicated by the setting levels of the commercial US systems used [25,33]. Therefore, they have not considered the real acoustic pressure distribution on the cell monolayer. In order to calculate the acoustic intensity, measurement of the acoustic parameters in the experimental conditions should be used. Standardization is necessary in order to avoid methodological differences between papers [14,18,22,30,34].

The quantification of several acoustic parameters of the two protocols was carried out in order to facilitate the comparison of our results (see Table 1, Table 2 and Table 3) with those published in the literature. Nevertheless, comparison between studies is difficult, as specific characteristics of the parameters are sometimes neglected (i.e., if it is the maximum or average value of intensity).

As an example of the discrepancies that could appear when comparing published results relating to cell cultures, the power of the transducer used in our experiments—measured using the force balance method (I_SATP_) in free propagation conditions—for the case of the LP signal protocol was 20 times less than the value calculated experimentally with the peak acoustic pressure data (I_SPTP_); see Table 1. This result highlights the need to measure the real acoustic pressure values applied to the cells during the experiments and not the acoustic pressure calculated with the acoustic intensity output, as indicated by the commercial equipment.

To ensure that no relevant heating is produced in the fluid, a thermocouple placed at 1 mm depth from the well bottom was used to register the temperature increase during the most intense sonication (VHP). The fluid temperature increased 4 °C from the initial value. It is well-known that the maximum temperature that a cell can withstand without being damaged is 45 °C, which supposes an increase of 8 °C from the initial culture temperature [35]. The culture warming finished immediately when the excitation pulse was turned off, so we can conclude that there was no heating that was harmful to the cells during sonication.

### 3.2. Effect of the Amplitude Variation of a Long US Pulse: LP Protocol

**Figure 6:**
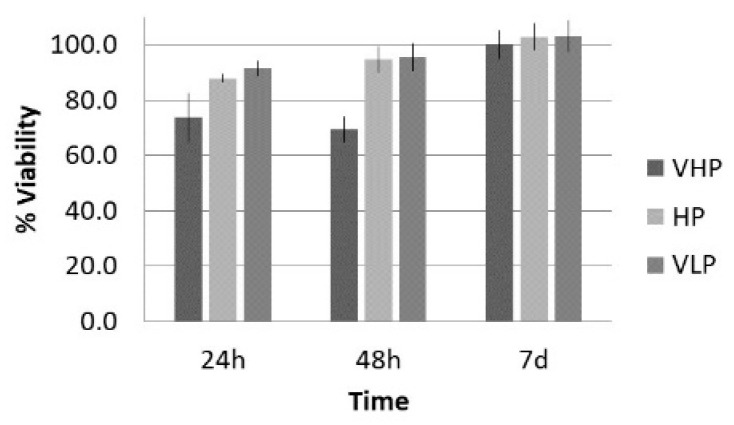
Viability (%), with respect to control, at 24 h, 48 h, and 7 d after treatment of fibroblasts with the long pulse protocol.

**Figure 7:**
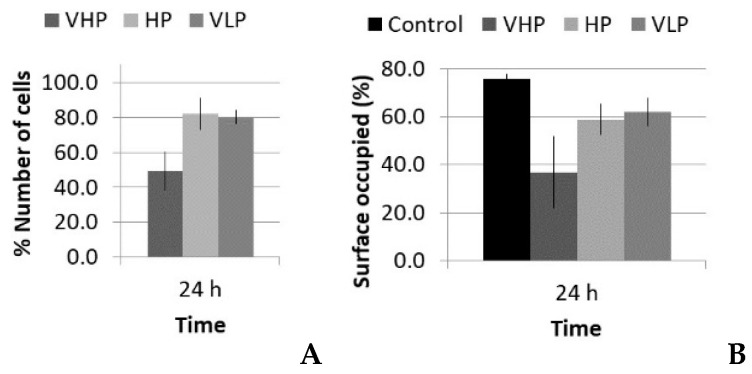
% (**A**) Number of cells and (**B**) surface occupied, with respect to control, 24 h after treatment with the long pulse protocol.

The data of the mean viability, number of cells, and surface occupied under the LP protocol while varying the pressure amplitude are shown in Figure 6 and Figure 7 for the groups listed in Table 1. In the case of viability and number of cells, the 100% value corresponds to control group. It was expected that the viability value increases with time as the cell population grows, resulting in a bigger metabolic rate, which the AlamarBlue^®^ assay evaluates (see Section 2.4). In the case of surface occupied, 100% represents the total culture well surface; we included the control data to facilitate comparison.

Lytic effects were observed in the VHP group. The surface occupied of the cells at 24 h was less than the original (37% vs. ≈50%). A 51% decrease in the number of cells was observed, which indicated 74% viability, with respect to the control. Image analysis revealed morphological changes in the highest acoustic intensity zones (see Table 4). Smaller fibroblasts with more and narrower prolongations were observed. These changes provide evidence of cell damage. The viability at 48 h was 69% lower than that of the control, indicating that necrosis was also induced. For these cases, we could not take images of the culture 48 h after sonication, as several overlapping cell sub-layers were present.

In the HP and VLP cases, there was a delay in the growth rate during the first 24 h (88% and 91%, respectively, compared with the control), a decrease in number of cells (18% and 20%, respectively, compared with the control), and a decrease in occupied surface (59% and 62%, with respect to 76% in the control group). This reduction was almost recovered at 48 h, with a metabolic rate close to that of the control (96% and 95%, respectively; see Figure 6). Lytic effects appeared, but no necrosis seemed to be induced by these two lower intensity treatments.

### 3.3. Effect of the Pulse Repetition Frequency Variation of a Short US: SP Protocol

**Figure 8:**
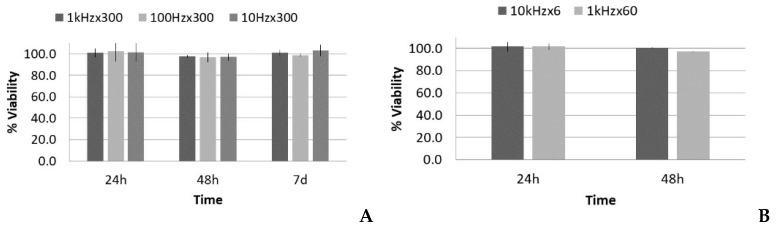
Viability (%), with respect to the control, 24 h, 48 h, and 7 d after treatment of fibroblasts with the short pulse protocol: (**A**) groups with the same total time of sonication (300 s) and (**B**) groups with the same total energy (60,000 pulses).

**Figure 9:**
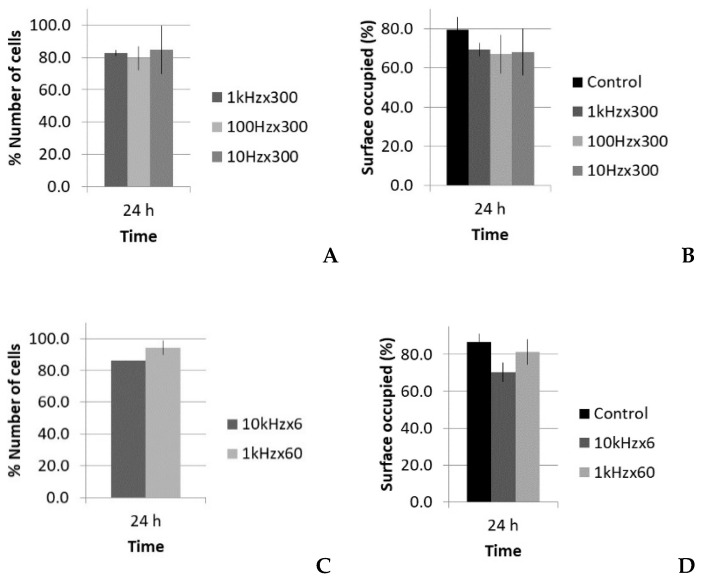
Number of cells and surface occupied (%), with respect to the control, 24 h after treatment of fibroblasts with the short pulse protocol: (**A**,**B**) groups with the same total time of sonication (300 s) and (**C**,**D**) groups with the same total energy (60,000 pulses).

There were two different situations for the five-cycle pulse: one case with increasing PRF and same total time of sonication (increasing number of total pulses), and another case with increasing PRF and proportional reduction of total time of sonication (i.e., with the same number of total pulses and same total energy). The acoustic parameters for these two SP groups are listed in Table 2 and Table 3, respectively. Following the analysis carried out in the LP case, data regarding the viability, number of cells, and occupied surface are shown in Figure 8 and Figure 9.

Figure 8A shows that there was no significant reduction of viability in all cases at the first 24 h post treatment, thus lytic effects were rejected. At 48 h, there was a slight reduction of viability, near 98–97%, which could suggest a small decrease in the growth rate, but it was in the statistical dispersion range of the measurements. After 7 days, all the groups had similar values as the control. All of the groups showed the same tendency, despite that fact that there was a factor of 100 difference in the number of pulses when the same sonication time was maintained (see Table 2). Figure 8B shows that there was also no significant reduction in viability.

Concerning the optical microscopy results (Figure 9), a decrease in the number of cells and occupied surface appeared at 24 h in all cases. In the first set of experiments (same sonication time, 300 s), the extent of cell reduction was similar in all groups (17%, 20%, and 15%), as well as the results for the occupied surface (69%, 67%, and 68%; Figure 9A,B); however, in the second set of experiments (same total energy), the reduction in cell number was higher for the highest PRF (14%; 10 kHz), and thus in the occupied surface (see Figure 9C,D).

Optical analysis in the two cases with equal total energy (10 kHz × 6 s and 1 kHz × 60 s; Table 3) showed differences in the number of binucleated fibroblasts. Table 5 shows the detected increase in binucleated fibroblasts. The relative number of binuclear cells seemed to not depend on the PRF, but instead on the number of pulses. 

## 4. Discussion

Quantitative measurement of the acoustic parameters was necessary to correlate the changes obtained at the cellular level. A review of conventional dosage methodologies revealed that proper quantification and design of the treatment protocols are needed. Our study revealed that relevant values may differ by a factor of up to 20, depending on the parameters considered. This highlights the need to measure the real acoustic pressure values that are applied to the cells during the experiments and not, as an example, the acoustic pressure calculated with the acoustic intensity output indicated by the commercial equipment used in the experiment.

Thermal effects were also dismissed, as a 4 °C increase over a short period of time (the duration of the sonication) was not considered enough to cause changes or damage to the cells, based on the literature [35]. To explore this aspect in experiments with higher or larger temperature variations, a specific study of a control group subjected to the same temperature conditions should be carried out. 

The radiation force estimation value for the I_SATP_ values used in our experiments (<1 W) was in the order of 10 Pa, which should not be enough to induce any effect in cells, as the magnitude order is near that for perfusion (0.1–4 Pa) [36]. Therefore, no cellular effects due to radiation forces were considered in our experiments.

**Figure 10:**
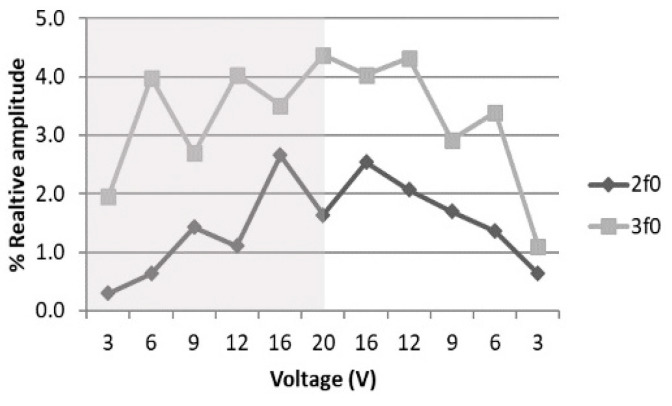
Dependence of relative amplitude of 2$f_{0}$ and 3$f_{0}$, with respect to $f_{0},\;$ with transducer excitation voltage variation when the voltage is increased (shaded region) or decreased (withe region).

Cavitation is a non-thermal mechanism, which has been proposed in the literature as being responsible for the effects we observed in the LP protocol. We used sono-spectroscopy to confirm or reject its presence [9,37,38,39,40]. A needle hydrophone was placed at 1 mm from the well bottom and the acquired signal was analyzed. Figure 10 shows the increase in the FFT absolute value of the 2$f_{0}$ and 3$f_{0}$ components as the transducer excitation voltage increased from 3 V_pp_ up to 20 V_pp_ for 200-cycle pulses with PRF of 1 kHz, like that used in the LP protocol (see Table 1). Despite the fact that the maximum voltage employed in the experiments was 30 V_pp_, the analysis ended at 20 V_pp_, in order to avoid signal distortion in the sensors owing to the use of a high voltage. If cavitation is detected at 20 V_pp_, it must also be present for higher values.

**Figure 11:**
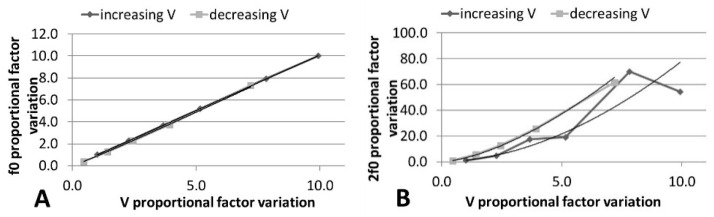
Variation of (**A**) $f_{0}$ and (**B**) 2$f_{0}$ in absolute FFT with linear increase in electrical voltage. Polynomic fitting is included.

The 3$f_{0}$ component is always higher than the 2$f_{0}$ component, and this behavior does not change with the signal time duration, such that the presence of cavitation was confirmed [41]. It has also been predicted that, for a non-linear process, $f_{0}$ increases as the driving voltage increases, while 2$f_{0}$ is proportional to the square of the voltage [42]. The analysis shown in Figure 11 and the polynomial fitting parameters given in Table 6 confirm this fact. When the voltage decreased from 20 V_pp_, the non-linear terms were higher for the same input; this hysteresis effect also provides evidence of cavitation [40].

The pressure amplitudes used in the LP protocol (P_max_, from 77 kPa up to 770 kPa) were enough to develop stable cavitation from the gas dissolved in the medium, in concordance with the calculations for the acoustic threshold (100 kPa) for the appearance of rectified diffusion [43]. The presence of 2$f_{0}$ and 3*f*_0_ harmonics, as shown above, confirmed this fact.

In the long pulse (LP) treatments, there were decreases in the population and viability after the first 24 h. In the VHP group, the number of cells at 24 h was even less than that of the original population. A lytic effect was supposed and, finally, confirmed because cavitation took place. The highest pressure amplitude levels (VHP) produced lysis and caused irreparable damage to the fibroblasts. Microscopy images confirmed that, in the zone of higher sonication intensities (the center of the transducer and left side; see Figure 3), the effect was more evident and dead cells were more commonly found (see Table 4). In the case of HP and VLP groups, the decrease in the growth rate at 24 h was considered to be related to necrosis mechanisms induced by cavitation owing to membrane disruption, chromosomal aberrations, and genomic damage [21,22,44,45,46]. This is in agreement with Feng Y et al., who suggested that, for very high intensities (I_SATA_ = 10 W/cm^2^), lysis is produced in minutes, whereas at lower intensities (I_SATA_ = 3 W/cm^2^), necrosis mechanisms are induced [19]. These values were in the same order as the maximum space peaks of our sound field (see Table 1). In the HP and VLP groups, the viability decrease was recovered in 48 h, matching the duplicative rate of this cell line [47].

Despite the initial decrease in the population, the damage was recovered in 7 days, such that the remaining fibroblasts had a higher metabolic rate. Biological responses due to mechanotransduction processes were promoted (e.g., increasing the secretion of growth factors, collagen synthesis, or calcium uptake). This was in concordance with previously reported works [14,23,24,25,26,27,29,48,49].

The pulse used in the SP treatments had an amplitude at least one order of magnitude less than the high-intensity US short pulses used in shock wave treatments (>10 MPa), in which harmful effects proportional to the number of shocks and time of sonication have been reported [50,51]. Interestingly, the PRF in shock wave clinic treatments is in the order of 1 Hz, thus the interpulse period is in the order of 1 s—much longer that that used in our SP treatment.

As seen in Figure 8, no significant reduction of viability in all SP treatments was found at the first 24 h. Based on the microscopy images, we also observed that no physical damage was caused to cells, thus lytic effects were rejected. Nevertheless, an increase in binuclear individuals was detected, which is a sign of genetic alterations (Table 5).

For the five-cycle SP treatment, cavitation was not detected. We found that a minimum of 10 cycles with 0.4 MPa pressure is needed for natural micronuclei to grow and to start oscillating in our experiment [52]. This result justifies that no lysis in SP treatment exists and, as a consequence, no decrease in viability was observed at 24 h (Figure 8).

In the first set of SP experiments (300 s sonication time), the cell reduction was similar in all groups (Figure 9A), as well as the decrease in the occupied surface (Figure 9B). Thus, the effect seems not be dependent on the number of pulses, as the PRF changed by a factor of 100. Previous authors have obtained no viability difference despite PRF variation [53], but viability decreases have been observed if the pulse is long enough to harm cells [18,54,55]. The question is as follows: What are the leading actions that govern the experiment, if the number of pulses does not increase the effect?

The explanation could be related to the fact that, after each pulse, the cells need time to sense and react, and the rate of stimulation has an influence [56]. The longer the time need to develop a reaction to the mechanical pulse, the bigger the effects of each pulse should be. As the total effect was similar in the three cases, it can be assumed that the effect was inversely proportional to the time between pulses. A similar tendency has been observed in an electropermeabilization study [57].

Comparing the two 1 kHz PRF cases (with 60 s or 300 s time sonication), the effect on proliferation seemed be proportional to the sonication time (see Figure 9A,C). In this case, under a longer treatment time with the same PRF (i.e., more pulses), there was a smaller number of cells. With this PRF, the leading parameter seemed to be the number of pulses; that is, the effect was accumulative. In this case, the combination of a net decrease in cells over time and better individual metabolic rate could explain that the viability was not affected.

However, in the second set of experiments (same total energy), the effect was more pronounced for the highest PRF (10 kHz) with only 6 s sonication time, with a reduction of 14% in the number of cells, compared with the 7% in the 60 s sonication case with a PRF of 1 kHz (Figure 9C). In this case, the number of pulses was the same, but the effect was bigger; that is, it seemed to not be accumulative. The answer may be that the time between pulses at a PRF of 10 kHz (0.1 ms) was smaller than the time needed for cells to develop mechanotransduction processes. The cell sensed the mechanical input, but it could not react properly, as occurred when we gave it time to react and develop reactions (i.e., under 1 kHz PRF). A cell blocking effect seems to appear at the cellular level, which decreases the cell growth rate. This is in concordance with cell physiology, as it is well-known that cell mechanotransduction processes take time in the order of one millisecond, the same time threshold we found in this experiment [58,59]. This was also confirmed by the optical analysis of binucleated cells, which revealed less anomalies in low PRF groups.

This work aimed to contribute to the study of the biophysical effects of US with intensities in the range of those used in therapeutic high intensity modalities (i.e., HIFU, with I > 1 KW/cm^2^) [6,60] and those used in physiotherapeutic applications (with I ≈ 1 W/cm^2^) [4,61,62]. To discuss the applicability of the results obtained in in vitro studies of US with intensity and/or acoustic pressure in this intermediate window to in vivo treatments, it should be noted that fibroblasts are immersed in the extracellular matrix, which makes up the connective tissue in an organism. A decrease in the number of cells, such as that observed in this work, can trigger tissue signals (i.e., release of growth factors), causing reactions in various cell lines (i.e., migration of satellite cells, monocytes, macrophages, and so on). The combination of these processes at the cellular level promotes repair mechanisms and accelerates the normal tissue recovery process. It is especially interesting to investigate these treatments in chronic lesions, where modification or even replacement of chronic tissue—mainly dysfunctional—with new cells having full capacity is part of the rehabilitation process.

However, it should be noted that the environment surrounding cells in a culture is not the same as that in vivo. Moreover, the tissues have different composition (i.e., tendons, ligaments, or muscles have a much greater protein content than skin or adipose tissue and, for that reason, they absorb more US energy). In the case of a long pulse, where cavitation is the main agent responsible for the observed effects, it is important to note that the probability of this phenomenon occurring in vivo varies according to the gaseous content of the tissue. It is well-known that, in general, the possibility of cavitation in vivo is low, but not null [63,64,65]. On the other hand, the introduction of contrast agents can significantly increase the likelihood of cavitation effects. In addition to composition, other factors, such as cell density (which is much higher in tissues than in cultures), also influence the effect achieved with US [66]. In the case of a short pulse, if we wish to have the same mechanical stimulation as that in an in vitro experiment, it should be considered that the attenuation of the wave when propagating in the medium must be taken into account in order to increase the acoustic pressure, if necessary. Changes in the acoustic diffraction due to the presence of interfaces between different tissues (i.e., between muscle and bone) should also be considered.

## 5. Conclusions

To obtain effects with US at the cellular level that alter repair processes in injured tissues, a certain sonication time is needed. With high amplitude pulses, above the in vivo cavitation threshold, harmful effects can arise in cells, which may trigger certain reactions (e.g., the release of more growth factors, migration, and so on) that cause further reactions in various cell lines. For this reason, it is particularly interesting to apply this type of treatment when the modification or replacement of tissue is desirable; for example, as occurs in chronic lesions or dysfunctional tissues.

The effects of the real transducer pattern and the finite dimensions of the culture plate wells on the acoustic pressure field distribution were studied. Standing wave formation and multiple pulse reflections can change the supposed acoustic intensity applied to the fibroblast cells dramatically when only the transducer output power is considered as the experimental reference value.

Two ultrasound dosage protocols, designed to study non-thermal effects in muscle skeleton diseases, were tested in an in vitro experiment using human fibroblasts. The major findings were as follows: (i) Long burst (200 excitation cycles/s) pulses with intensity from 0.19 W/cm^2^ up to 19.25 W/cm^2^ promoted lytic effects owing to stable cavitation. The cultures showed decreases in viability, number of cells, and occupied surface of the cells’ monolayer, as well as showing cell morphology changes, as the intensity increased; (ii) High amplitude short bursts (five excitation cycles, 33.1 W/cm^2^) promoted mechanotransduction reactions. No cavitation was detected in the cultures. When decreasing the frequency of the pulses (PRF) from 1 kHz down to 10 Hz, an increase in the effect appeared, which seemed linear with the time between pulses. The longer the time between pulses, the stronger the effect. At 1 kHz, the effect was linear with the number of pulses. The more pulses, the stronger the effect. When increasing the frequency of the pulses (PRF) from 1 kHz to 10 kHz, the number of cells decreased and the relative number of binucleated cells increased. The mechanotransduction channels seemed to be blocked, as the time between pulses was not long enough to permit the complete development of the associated cell reaction, thus affecting the growth cell rate.

These two protocols can be applied to animal tests, separately or combined. The LP, which promotes cavitation, lysis, and necrosis, should be used for tissue deconstruction, whereas the SP protocols would be better used for tissue generation purposes.

## Figures and Tables

**Table 1:** Sonication parameters for the long pulse protocols (200 cycles of increasing amplitude P and, therefore, increasing energy density ρ): very high pressure (VHP), high pressure (HP), and very low pressure (VLP).

	$V_{pp}$ V	PRF Hz	$P_{max}$ kPa	$I_{SPTP}$ W/cm^2^	$I_{SATP}$ mW/cm^2^	$\rho_{E}$ mJ/mm^2^	$\rho_{E\;total}$ mJ/mm^2^
VHP	30	10^3^	770	19.25	880	7.21 × 10^−3^	2163
HP	6	10^3^	154	0.77	30	2.88 × 10^−4^	86.4
VLP	3	10^3^	77	0.19	7	0.72 × 10^−3^	21.6

**Table 2:** Sonication parameters for the short pulse protocols (five cycles of high amplitude P and the same energy density ρ in all cases), with the same total time of sonication (300 s) and variable pulse repetition frequency (PRF; 1 kHz, 100 Hz, or 10 Hz), such that the total energy provided to the cells increases.

**PRF × Time**	$V_{pp}$ V	PRF Hz	$P_{max}$ kPa	$I_{SPTP}$ W/cm^2^	$I_{SATP}$ mW/cm^2^	$\rho_{E}$ mJ/mm^2^	$\rho_{E\;total}$ mJ/mm^2^
1 kHz × 300 s	120	10^3^	1.01	33.1	16.2	1.48 × 10^−3^	444
100 Hz × 300 s	120	10^2^	1.01	33.1	16.2	1.48 × 10^−3^	44.4
10 Hz × 300 s	120	10	1.01	33.1	16.2	1.48 × 10^−3^	4.4

**Table 3:** Sonication parameters for the short pulse protocols (five cycles of high amplitude P and the same energy density ρ in all cases) with the same total energy in all cases; that is, the same total number of pulses over the total period of sonication: 6 s or 60 s for the pulse repetition frequencies (PRFs) of 10 kHz and 1 kHz, respectively.

**PRF × Time**	$V_{pp}$ V	PRF Hz	$P_{max}$ kPa	$I_{SPTP}$ W/cm^2^	$I_{SATP}$ mW/cm^2^	$\rho_{E}$ mJ/mm^2^	$\rho_{E\;total}$ mJ/mm^2^
10 kHz × 6 s	120	0.1	1.01	33.1	16.2	1.48 × 10^−3^	88.8
1 kHz × 60 s	120	1	1.01	33.1	16.2	1.48 × 10^−3^	88.8

**Table 4:** Comparative images of the cultures 24 h post-treatment.

Control	VHP
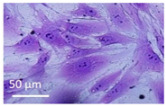	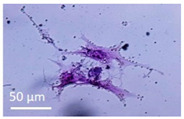
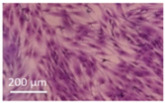	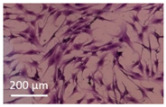

**Table 5:** Number of binucleated cells in the case of short pulse treatment with the same energy. Left: image of a binucleated fibroblast.

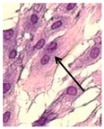	**Protocol**	**Total Images**	**Binuclei**	**(Binuc/Image)**
	Control	9	10	1.1
	10 kHz × 6 s	10	23	2.3
	1 kHz × 60 s	10	21	2.1

**Table 6:** Polynomial fitting parameters of curves in Figure 11.

	y = ax^n^ ↑V	*R*^2^ ↑V	y = ax^n^ ↓V	*R*^2^ ↓V
*f*_0_	y = 0.9949x^1.0068^	1	y = 0.8942x^1.0549^	0.9997
2*f*_0_	y = 1.1022x^1.8518^	0.9674	y = 3.0569x^1.5536^	0.9985

## Data Availability

Not applicable.
